# Preparation of Large Monodisperse Vesicles

**DOI:** 10.1371/journal.pone.0005009

**Published:** 2009-04-06

**Authors:** Ting F. Zhu, Jack W. Szostak

**Affiliations:** 1 Howard Hughes Medical Institute and Department of Molecular Biology, Massachusetts General Hospital, Boston, Massachusetts, United States of America; 2 Harvard-MIT Division of Health Sciences and Technology, Massachusetts Institute of Technology, Cambridge, Massachusetts, United States of America; Johns Hopkins School of Medicine, United States of America

## Abstract

Preparation of monodisperse vesicles is important both for research purposes and for practical applications. While the extrusion of vesicles through small pores (∼100 nm in diameter) results in relatively uniform populations of vesicles, extrusion to larger sizes results in very heterogeneous populations of vesicles. Here we report a simple method for preparing large monodisperse multilamellar vesicles through a combination of extrusion and large-pore dialysis. For example, extrusion of polydisperse vesicles through 5-µm-diameter pores eliminates vesicles larger than 5 µm in diameter. Dialysis of extruded vesicles against 3-µm-pore-size polycarbonate membranes eliminates vesicles smaller than 3 µm in diameter, leaving behind a population of monodisperse vesicles with a mean diameter of ∼4 µm. The simplicity of this method makes it an effective tool for laboratory vesicle preparation with potential applications in preparing large monodisperse liposomes for drug delivery.

## Introduction

Vesicles are closed bilayer membranes that encapsulate an aqueous compartment. Fatty acid vesicles have been studied as a model system for primitive cellular membranes at the origin of life [1], [2]. Phospholipid vesicles (liposomes), which are more stable under physiological conditions, have been widely used for drug delivery [3]. There are many advantages to being able to work with monodisperse vesicle preparations. In studies of vesicle growth, monodisperse vesicle preparations allow for the detection of changes in vesicle size by light scattering or by fluorescence microscopy [2], [4]. The size distribution of vesicles is a crucial factor in determining the efficacy of drug delivery [5]. The accumulation of liposomes in tumors is size-dependent, as tumor capillaries have larger pores (100 to 700 nm in diameter) than normal blood vessels (typically<50 nm). Thus liposomes between 90 and 200 nm in diameter can selectively penetrate tumor capillaries [6]. The drug release profile from liposomes *in vivo* has also been shown to be size-dependent [7]. For inhaled liposomal drug delivery, the ideal liposome size is between 1 and 3 µm [8]–[10], because particles in this size range can be delivered into the deep lung more effectively and avoid phagocytic clearance from the lung periphery [9].

Vesicles formed by resuspending a dried film of lipids are highly heterogeneous in size [11], [12]. Various methods have been used to directly prepare monodisperse vesicles, including vesicle extrusion and vesicle formation from double emulsions [13]–[15], or to purify monodisperse vesicles from heterogeneous populations, such as gel filtration [16] and high performance size exclusion chromatography (HPSEC) [17]. Extrusion is often used for making relatively homogeneous vesicles in the size range between 50 and 100 nm [18], [19]. This approach takes advantage of the fact that when vesicles are forced through membrane pores smaller than their diameter, they break down into smaller vesicles closer to the pore size [20]. While effective in making small (50 to 100 nm) monodisperse vesicles, this technique does not eliminate vesicles smaller than the membrane pore size. Because of the initial population heterogeneity, vesicles extruded through pores larger than ∼200 nm remain quite heterogeneous. HPSEC has been used to purify vesicles prepared by extrusion [12], [17], but is only capable of producing monodisperse vesicles smaller than 300 nm because of limitations on the available pore sizes of the column gel. Double emulsions (e.g., water/oil/water emulsions) prepared using a microfluidic device [13]–[15] can be used to make giant monodisperse vesicles, but the process is complicated, and contamination by the oil phase remains an issue for drug delivery applications.

**Figure 1:**
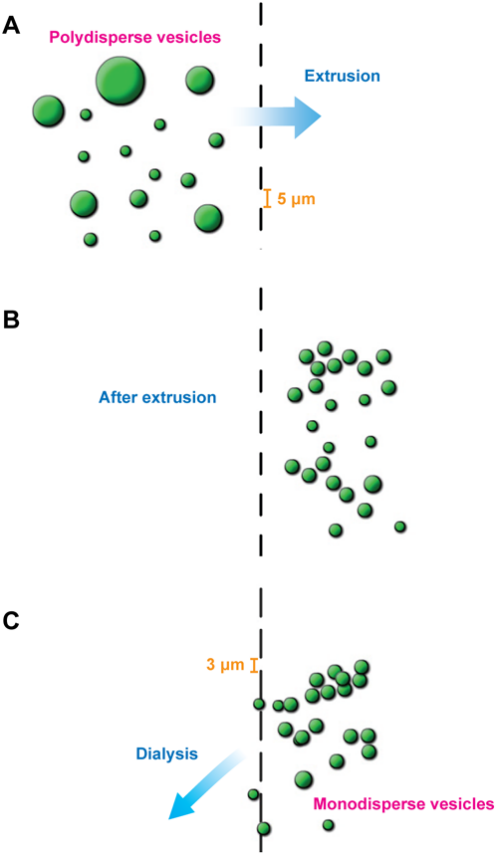
Schematic diagram of vesicle extrusion-dialysis. (A, B) Extrusion of polydisperse vesicles through 5-µm-diameter pores eliminates vesicles larger than 5 µm in diameter. (C) Dialysis of extruded vesicles against 3-µm-pore-size polycarbonate membranes eliminates vesicles smaller than 3 µm in diameter, leaving behind a population of monodisperse vesicles (see also Movie S1).

We have developed a simple method for preparing monodisperse vesicles through a combination of extrusion and large-pore dialysis. We use polycarbonate track-etched membranes with large pore sizes (submicron to several microns in diameter) first for extrusion, and then for dialysis to remove vesicles smaller than the membrane pores. For example, extruding through a polycarbonate membrane with 5-µm-diameter pores and dialyzing using membranes with 3-µm-diameter pores results in a vesicle population between 3–5 µm in diameter (Figure 1; see also Movie S1).

## Methods

### Materials

Fatty acids and fatty acid derivatives were obtained from Nu-chek Prep (Elysian, MN). Fluorescent dyes were obtained from Molecular Probes, Inc. (Eugene, OR). Oleate (C18:1) vesicles were prepared by resuspending a dried film of oleic acid in 0.2 M Na-bicine (*N,N*-Bis(2-hydroxyethyl)glycine, Sigma-Aldrich, St. Louis, MO) containing 2 mM HPTS (8-hydroxypyrene-1,3,6-trisulfonic acid trisodium salt, a water-soluble, membrane-impermeable fluorescent dye) at pH 8.5, to a final concentration of 10 mM oleic acid in buffer. The vesicle suspension was vortexed briefly, and tumbled overnight. POPC (1-palmitoyl-2-oleoyl-*sn*-glycero-3-phosphocholine) vesicles were prepared by resuspending a dried film of POPC in 0.2 M Na-bicine containing 2 mM HPTS at pH 8.5, to a final concentration of 10 mM POPC in buffer. For oleate vesicles, the washing buffer for dialysis was prepared by resuspending 10 mM oleic acid in 0.2 M Na-bicine buffer at pH 8.5 but without fluorescent dye, to maintain the concentration of oleate acid above its cac (critical aggregation concentration) and avoid vesicle dissolution. Dialysis of POPC vesicles was performed using 0.2 M Na-bicine at pH 8.5 without POPC vesicles, since the cac of POPC vesicles is so low that vesicle dissolution was not a concern.

### Vesicle extrusion and large-pore dialysis

Large-pore dialysis cassettes were made by modification of commercially available 500 µl dialysis cassettes (Pierce, Rockford, IL). The original membranes on the cassette were replaced with polycarbonate track-etched membranes (Whatman, United Kingdom). Such membranes have sharply defined pore sizes, and have been used for vesicle extrusion in previous studies [2], [4], [21], [22]. About 400 µl of extruded vesicles encapsulating HPTS were loaded onto the center of a modified dialysis cassette, after which the cassette was closed with metal clamps. A volume of ∼30 ml washing buffer was used in each round of dialysis, which just submerged the horizontally placed dialysis cassette in a 150 ml beaker. The beaker was gently agitated on a table-top shaker at 60 rpm. The first 5–6 rounds of dialysis were for 5–10 min each, after which the free dye in the vesicle suspension was adequately eliminated. At least 6 more rounds of dialysis (each for 2 hrs minimum, one of which was overnight) were performed to eliminate vesicles smaller than the membrane pores. The vesicle sample was retrieved with a pipette tip by breaking the polycarbonate membrane after dialysis. For oleate vesicles, the resultant vesicle population contained large monodisperse vesicles encapsulating fluorescent dye and smaller vesicles from the washing buffer that were dye-free (since they are not fluorescent, their presence does not affect the imaging and the counting of large dye-labeled vesicles by fluorescence microscopy). Extrusion and large-pore dialysis of POPC vesicles were performed using the same method. The monodisperse POPC vesicle population did not contain any dye-free vesicles as the washing buffer did not contain any POPC vesicles.

### Vesicle size distribution and lamellarity

To determine the size distribution of resuspended oleate vesicles before and after the removal of small vesicles, oleate vesicles (containing 2 mM HPTS) were extruded through a polycarbonate membrane with 5-µm-diameter pores and dialyzed using conventional dialysis membranes (10 kDa cutoff) to eliminate the free dye (for the purpose of imaging) from the vesicle suspension. Vesicles were imaged using a Nikon TE2000S inverted epifluorescence microscope with extra long working distance (ELWD) objective lenses. Vesicle sizes were analyzed by Phylum Live software (Improvision, Lexington, MA). The size distribution of POPC vesicles was determined using the same method. To study the lamellarity of the monodisperse oleate and POPC vesicles, confocal images were taken using a Leica SP5 AOBS scanning laser confocal microscope with Leica acquisition software (Leica, Germany). The vesicles were labeled with a membrane-anchored fluorescent dye, Rh-DHPE (Lissamine™rhodamine B 1,2-dihexadecanoyl-*sn*-glycero-3-phosphoethanolamine; excitation at 560 nm, emission at 586 nm).

## Results

**Figure 2:**
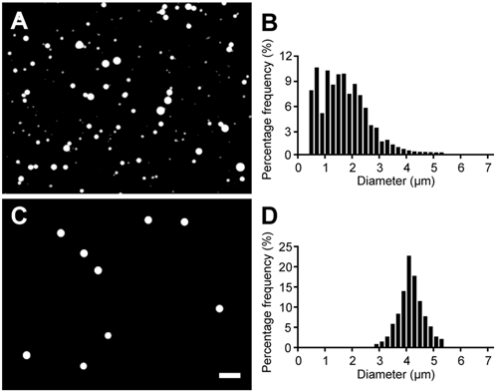
Preparation of large monodisperse oleate vesicles. (A) Oleate vesicles (containing 2 mM HPTS, in 0.2 M Na-bicine, pH 8.5) after being extruded through 5-µm-diameter pores, and (B) the corresponding size distribution, shown as percent per 0.2 µm bin (vesicles between 0–0.4 µm in diameter were not counted). (C) After 12 rounds of dialysis using membranes with 3-µm-diameter pores, and (D) the corresponding size distribution. Scale bar, 10 µm.

Oleic acid vesicles, after being extruded through a 5-µm-pore-size membrane, as shown in Figure 2A, were almost all less than 5 µm in diameter. As expected, the vesicles were highly heterogeneous in size, with the bulk of the vesicles being smaller than 3 µm in diameter. The size distribution is shown in Figure 2B. A large number of vesicles in the size range between 0 and 0.4 µm were too small to be accurately measured, and therefore were not counted. The fraction of 3–5 µm vesicles within this population was estimated as ∼4%. After 12 rounds of dialysis with 3-µm-pore-size membranes, vesicles smaller than 3 µm in the population were almost entirely eliminated, resulting in a relatively narrow population size distribution between 3 and 5 µm in diameter (Figure 2C and 2D). The average diameter was 4.2 µm, with a standard deviation of ±15%. These vesicles contain ∼40% of the total encapsulated volume of the original population, with the remaining ∼60% of the encapsulated contents lost due to removal of the smaller vesicles. The recovered 3–5 µm vesicles retained the encapsulated fluorescent dye and remained monodisperse for at least several weeks.

**Figure 3:**
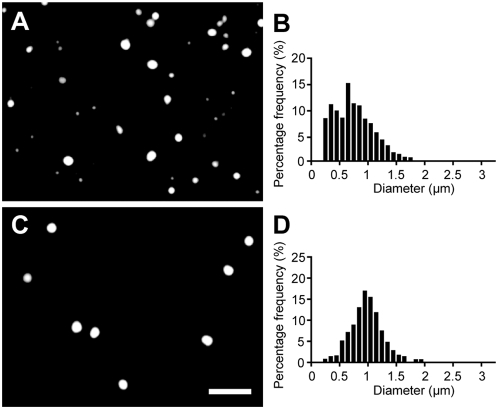
Preparation of monodisperse POPC vesicles. (A) POPC vesicles (containing 2 mM HPTS, in 0.2 M Na-bicine, pH 8.5) after being extruded through 1-µm-diameter pores, and (B) the corresponding size distribution, shown as percent per 0.1 µm bin. (C) After 12 rounds of dialysis using membranes with 0.8-µm-diameter pores, and (D) the corresponding size distribution. Scale bar, 5 µm.

**Figure 4:**
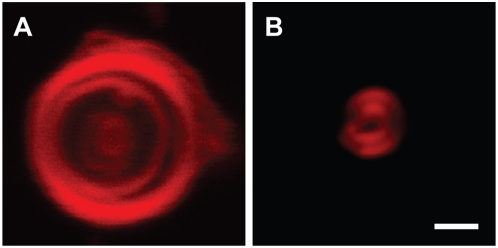
Confocal images of oleate and POPC vesicles prepared by extrusion-dialysis. (A) Confocal image of a multilamellar oleate vesicle after extrusion-dialysis (0.2 mol % Rh-DHPE, in 0.2 M Na-bicine, pH 8.5). (B) Confocal image of a multilamellar POPC vesicle after extrusion-dialysis (0.2 mol % Rh-DHPE, in 0.2 M Na-bicine, pH 8.5). Though the vesicle is small (∼0.5 µm in radius, approaching the resolution limit of the confocal microscope), the image clearly indicates the presence of an internal membrane. Scale bar, 1 µm.

We have used essentially the same procedures to prepare POPC vesicles of a different size range. Dye-labeled POPC vesicles extruded through a 1-µm-pore-size membrane, as shown in Figure 3A and 3B), were heterogeneous in size. After dialyzing with 0.8-µm-pore-size membranes for 12 rounds, as shown in Figure 3C and 3D), vesicles smaller than 0.8 µm were significantly reduced. The average final diameter was 1.0 µm with a standard deviation of ±30%. Thus the preparation of POPC vesicles between 0.8 and 1 µm in diameter by the extrusion-dialysis method created a less monodisperse population of vesicles than we obtained for larger (3 to 5 µm in diameter) oleate vesicles. As shown in Figure 4, both the oleate and POPC vesicles prepared by extrusion-dialysis were multilamellar. This is as expected, since the vesicles were initially formed by the resuspension of dried lipid films, which is known to produce predominately multilamellar vesicles with variable numbers of internal membranes.

## Discussion

The extrusion-dialysis method for preparing monodisperse vesicles that we have described is a hybrid preparation/purification method in which extrusion is used to prepare vesicles that are smaller than a given size, and then dialysis is used to eliminate vesicles smaller than a desired size threshold. This approach can be used to produce vesicles of different size ranges and with different lipid compositions. Since the extrusion-dialysis procedures are independent from how the vesicles are initially prepared (in the current study, via the resuspension of dried lipid films), this method should in principle be applicable to vesicles made in different ways, and to initial populations of vesicles with different lipid compositions, size ranges, and lamellarity. It may even be possible to develop a similar method to purify monodisperse solid particles from a population of polydisperse particles by removing particles above a size threshold by membrane filtration, followed by dialysis to eliminate particles below a smaller size threshold.

This method is most suitable for preparing large (several microns) monodisperse vesicles. This is convenient for applications involving fluorescence microscopy, because vesicles of several microns are ideal for imaging, as the optical resolution limit is ∼0.25 to 0.5 µm for conventional microscopes. Standard methods for preparing monodisperse vesicles, such as extrusion, are typically used to produce vesicles of <200 nm in diameter, thus the extrusion-dialysis method is an aptly complementary method. Though intravenous drug delivery typically uses liposomes smaller than 1 µm, certain clinical applications such as inhaled liposomal drug delivery require larger liposomes of several microns in diameter [8]–[10].

In addition to size control, control over the lamellarity of vesicles is also important for many applications. Since the method we used for initial vesicle formation (resuspension of dried lipid films) is known to produce predominately multilamellar vesicles, the monodisperse vesicles resulting from extrusion-dialysis were also multilamellar, as expected. Whether the extrusion-dialysis method can be applied to purify unilamellar vesicles prepared by other vesicle formation methods, such as electroformation, should be examined in future studies. Monodisperse multilamellar fatty acid vesicles are useful in origin-of-life studies as models of primitive cellular membranes, since vesicles that formed spontaneously in early earth environments (e.g., by the resuspension of dry fatty acid films or by the acidification of concentrated solutions of micelles) would be likely to be large and multilamellar [2], [23]. Monodisperse multilamellar phospholipid vesicles may find applications in drug delivery, as the high membrane-to-volume ratio would allow them to carry more lipophilic drugs in their bilayer membranes.

Our experience indicates that this preparation/purification method is not very sensitive to changes in the operating parameters (duration of dialysis, etc.) and thus should be easy to reproduce using similar or modified methods. Practical applications of this method may currently be constrained by the overall duration of the procedures (∼24 hr total), and by the low yield of monodisperse vesicles. On the other hand, the simplicity and versatility of the method should make large-scale preparation possible in practical applications.

## Supporting Information

Movie S1 Movie S1. Schematic movie of vesicle extrusion-dialysis. (QuickTime format; 6 MB) Extrusion of polydisperse vesicles through 5-µm-diameter pores eliminates vesicles larger than 5 µm in diameter. Dialysis of extruded vesicles against 3-µm-pore-size polycarbonate membranes eliminates vesicles smaller than 3 µm in diameter, leaving behind a population of monodisperse vesicles.
(6.30 MB MOV)
